# Comparative Genomics Analysis Provides New Insight Into Molecular Basis of Stomatal Movement in *Kalanchoë fedtschenkoi*

**DOI:** 10.3389/fpls.2019.00292

**Published:** 2019-03-13

**Authors:** Robert C. Moseley, Gerald A. Tuskan, Xiaohan Yang

**Affiliations:** ^1^Department of Biology, Duke University, Durham, NC, United States; ^2^Biosciences Division, Oak Ridge National Laboratory, Oak Ridge, TN, United States; ^3^The Center for Bioenergy Innovation, Oak Ridge National Laboratory, Oak Ridge, TN, United States; ^4^The Bredesen Center for Interdisciplinary Research and Graduate Education, The University of Tennessee, Knoxville, Knoxville, TN, United States

**Keywords:** *Kalanchoë fedtschenkoi*, stomatal movement, crassulacean acid metabolism, drought, rescheduled gene expression, *Arabidopsis thaliana*, *Solanum lycopersicum*

## Abstract

CO_2_ uptake and water loss in plants are regulated by microscopic pores on the surface of leaves, called stomata. This enablement of gas exchange by the opening and closing of stomata is one of the most essential processes in plant photosynthesis and transpiration, affecting water-use efficiency (WUE) and thus drought susceptibility. In plant species with crassulacean acid metabolism (CAM) photosynthesis, diel stomatal movement pattern is inverted relative to C_3_ and C_4_ photosynthesis species, resulting in much higher WUE and drought tolerance. However, little is known about the molecular basis of stomatal movement in CAM species. The goal of this study is to identify candidate genes that could play a role in stomatal movement in an obligate CAM species, *Kalanchoë fedtschenkoi.* By way of a text-mining approach, proteins were identified in various plant species, spanning C_3_, C_4_, and CAM photosynthetic types, which are orthologous to proteins known to be involved in stomatal movement. A comparative analysis of diel time-course gene expression data was performed between *K. fedtschenkoi* and two C_3_ species (i.e., *Arabidopsis thaliana* and *Solanum lycopersicum*) to identify differential gene expression between the dusk and dawn phases of the 24-h cycle. A rescheduled catalase gene known to be involved in stomatal movement was identified, suggesting a role for H_2_O_2_ in CAM-like stomatal movement. Overall, these results provide new insights into the molecular regulation of stomatal movement in CAM plants, facilitating genetic improvement of drought resistance in agricultural crops through manipulation of stomata-related genes.

## Introduction

Crassulacean acid metabolism (CAM) is a plant adaptation that involves a carbon concentrating mechanism that is based on a temporal separation of CO_2_ fixation (Ehleringer and Monson, 1993), which is facilitated by the inverted day/night pattern of stomatal closing and opening in comparison with C_3_ or C_4_ photosynthesis species (Males and Griffiths, 2017). Specifically, CAM species open their stomata during the night allowing for uptake of atmospheric CO_2_, which is converted into malate for storage in the vacuole. During the day, CO_2_ is released from malate while stomata are closed, resulting in CO_2_ accumulation around Rubisco for normal photosynthetic processes (Ehleringer and Monson, 1993). Additionally, the inversion of stomatal movement is an important drought avoidance/tolerance mechanism in CAM photosynthesis plants, by which water loss caused by evapotranspiration is decreased and consequently water-use efficiency is increased. Engineering of these traits into non-CAM species has great potential for genetic improvement of drought resistance in crops, which requires a deep understanding of the molecular mechanisms underlying stomatal movement in CAM photosynthesis plants (Borland et al., 2014; Yang et al., 2015).

**FIGURE 1:**
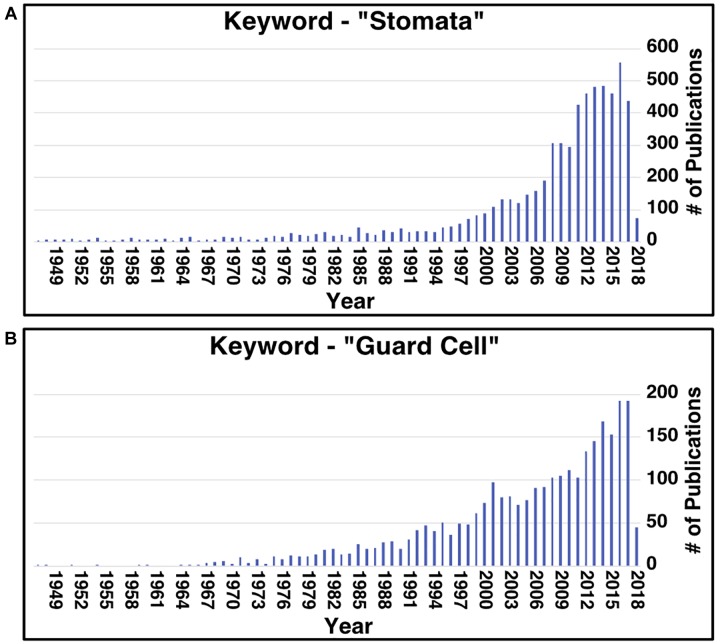
Number of publications per year related to stomata and guard cell research since 1949. Results of searching the PubMed database using the key words “stomata” **(A)** and “guard cell” **(B)**.

Aside from stomata research in CAM, research on stomata, in general, has recently increased (Figure 1). In all plants, stomata play essential roles in controlling water losses caused by transpiration and CO_2_ uptake for photosynthesis, modulating the transpiration-driven water flow through the soil-plant-atmosphere continuum, and plant adaptation to changing environmental conditions and stresses (Damour et al., 2010). Understanding stomatal development, movement, and patterning can facilitate engineering efforts to improve these traits. However, two issues today hinder the pace of advancement in stomatal research. Firstly, most work on these stomatal processes has been conducted in the model C_3_ plant *Arabidopsis thaliana*. This can present an issue when wanting to transfer the knowledge gained from these studies to another species as the genes involved in these processes can vary in regulation and/or function, as seen in CAM species (Abraham et al., 2016; Males and Griffiths, 2017; Yang et al., 2017), and in the grass species *Brachypodium distachyon* (Raissig et al., 2016, 2017). Secondly, homology between protein sequences can help infer protein function (Clark and Radivojac, 2011), but the issue is that many proteins’ functional information is missing from annotation databases or is hidden in the scientific literature. For instance, a protein of interest could be homologous to a protein characterized to function in a stomata-related process, but this information is not known because the characterized protein lacks the gene ontology (GO) annotation.

There are text-mining tools that can be used to search for information about similar proteins by combining BLAST searches with links to articles from certain databases (Gilchrist et al., 2008; Li et al., 2013; Jaroszewski et al., 2014). However, these tools do not search the literature, therefore, limiting their analysis. Recently, a new web-based tool called PaperBLAST (Price and Arkin, 2017), was developed to combine sequence-homology searches and text-mining of linked articles for predicting a protein’s function. PaperBLAST has potential to alleviate the two issues facing stomatal research described above, and therefore aid in discovering molecular mechanisms behind inverted stomatal movement seen in CAM species.

In this study, we first identified underexplored stomatal-related genes that contain no stomata-related annotations but have been linked to stomatal-related processes using PaperBLAST. Next, we identified genes that could be involved in the inverted stomatal movement in CAM plants through a comparative analysis of time-course gene expression data between an obligate CAM photosynthesis species (*Kalanchoë fedtschenkoi*) and two C_3_ photosynthesis species (*A. thaliana* and *Solanum lycopersicum*). Our research greatly expanded the catalog of stomata-related genes in plants, providing a resource for future experimental work in stomatal research of non-model species, and more importantly, providing gene targets for engineering CAM-like traits into non-CAM species.

## Materials and Methods

### Ortholog Groups and Phylogenetic Analysis

Ortholog groups consisting of proteins from 26 plant species were obtained from Yang et al. (2017). For phylogenetic analysis, protein sequence alignments were created using the web-based MUSCLE program^1^ with default parameters (Katoh and Standley, 2013; McWilliam et al., 2013; Li et al., 2015). Phylogenetic trees were generated from the protein sequence alignments using the web-based IQ-Tree program^2^ with default parameters (Trifinopoulos et al., 2016).

### Gene Ontology Analysis of Protein Sequences

Gene ontology terms for *A. thaliana* and *K. fedtschenkoi* genes were taken from the gene annotation information downloaded from Phytozome v12.1^3^ (Goodstein et al., 2012). The right-sided hypergeometric enrichment test at medium network specificity in ClueGO (Bindea et al., 2009) was used to identify the biological processes over-represented in individual gene sets. The Holm-Bonferroni step-down method (Holm, 1979) was performed for *p*-value correction. The minimum and maximum thresholds for selected GO-tree levels were 3 and 8, respectively, while individual clusters were required to include no less than 3% of genes associated with each GO term. To minimize GO-term redundancy, GO-term fusion and grouping settings were selected and the representative term for each functional cluster was determined as the term enriched at the highest level of significance. The GO terms with *p*-values less than or equal to 0.05 were considered significantly enriched.

### Data-Mining Using PaperBLAST

To include a variety of evolutionary lineages, the proteomes of 13 representative plant species including *Amborella trichopoda* (basal angiosperm, C_3_ photosynthesis), *Ananas comosus* (monocot, CAM photosynthesis), *A. thaliana* (dicot, C_3_ photosynthesis), *B. distachyon* (monocot, C_3_ photosynthesis), *K. fedtschenkoi* (dicot, CAM photosynthesis), *Mimulus guttatus* (dicot, C_3_ photosynthesis), *Musa acuminata* (monocot, C_3_ photosynthesis), *Oryza sativa* (monocot, C_3_ photosynthesis), *Phalaenopsis equestris* (monocot, CAM photosynthesis), *Setaria italica* (monocot, C_4_ photosynthesis), *S. lycopersicum* (dicot, C_3_ photosynthesis), *Sorghum bicolor* (monocot, C_4_ photosynthesis), and *Vitis vinifera* (dicot, C_3_ photosynthesis) were downloaded from the PLAZA 4.0 database (Van Bel et al., 2017; Supplementary Table S1). Each protein sequence was used as a search query in PaperBLAST (Price and Arkin, 2017) using an *E*-value threshold of 1e-3. The subsequent HTML files contain various information on the homologous proteins, such as functional information, article name, and text snippets from articles containing the homologous protein’s IDs. The HTML files were collected and parsed through searching for any presence of stomata-related keywords (i.e., “stomata” and “guard cell”) in each PaperBLAST hit’s affiliated data using in-house python scripts. Any homologous proteins that did not contain the keywords in their related data were filtered out. The relevant extracted data for each species are summarized in Supplementary Table S2.

### Analysis of Time-Course Gene Expression Data

The diel expression data for *K. fedtschenkoi* were taken from Yang et al. (2017), which were generated from transcriptome-sequencing (RNA-Seq) of whole leaf tissue samples collected at 2-h intervals from plants grown under a 12-h light/12-h dark cycle. The diel expression data for *A. thaliana* were taken from Mockler et al. (2007), which were generated from samples collected at 4-h intervals from plants grown under a 12-h light/12-h dark cycle. Raw RNA-Seq reads for *S. lycopersicum* were taken from the DDBJ Sequence Read Archive^4^ under the accession numbers DRA003529 and DRA0035530, which were generated from whole-leaf samples collected every 2 h from plants grown in a 10-h light/14-h dark cycle (Higashi et al., 2016). The *S. lycopersicum* raw sequencing reads were quality checked by FastQC (Andrew, 2014), trimmed using Trimmomatic v0.36 (Bogler et al., 2014) with default parameters, and quality checked again by FastQC. The trimmed *S. lycopersicum* RNA-Seq reads were mapped to the *S. lycopersicum* iTAG v2.3 reference genome using TopHat2 v1.0.1 (Kim et al., 2013) using default parameters. Read counts were computed using Cufflink v2.2.1 (Trapnell et al., 2012) and fragments per kilobase of transcript per million mapped reads (FPKM) were calculated. Processing of all raw sequencing data was performed using Kbase (Arkin et al., 2018). Genes with an average FPKM less than 0.01 and with less than half of their sampling points having an FPKM equal to zero were filtered out from the *K. fedtschenkoi* and *S. lycopersicum* expression data sets. Following previous works utilizing the *A. thaliana* time course transcriptome dataset (Abraham et al., 2016; Yang et al., 2017; Moseley et al., 2018), the *A. thaliana* data was adjusted to arrive at expression profiles on the same time scale as *K. fedtschenkoi* and *S. lycopersicum* gene expression data. Specifically, the cubic interpolation algorithm in the pandas Python library^5^ was used to simulate the *A. thaliana* gene expression data at additional time points so that all three data sets consisted of sampling points spaced every 2 h over a 24-h period.

### Comparative Analysis of Gene Expression

Comparative analysis of transcript expression between the time-windows dusk and dawn was performed as described by Yang et al. (2017). For both *K. fedtschenkoi* and *A. thaliana*, the dusk-window covers 10, 12, and 14 h after the starting of the light period and the dawn-window covers 22, 24, and 2 h after the starting of the light period (Supplementary Figure S1). For *S. lycopersicum*, which was grown under a slightly different light regime than *K. fedtschenkoi* and *A. thaliana*, the dusk-window covers 8, 10, and 12 after the starting of the light period, and the dawn-window covers 22, 24, and 2 h after the starting of the light period (Supplementary Figure S1). For each species, any genes with negative gene expression levels were removed and expression data for each gene was normalized as described in Yang et al. (2017). A two-column matrix was created, where rows represented genes, one column represented the sum of all transformed dusk time points, and the other column represented the sum of all transformed dawn time points. For each gene, the right-tailed Fisher Exact Test was used to determine if that gene’s expression was enriched in dusk, dawn, or neither, according to the contingency tables described in Yang et al. 2017; (Supplementary Figure S1). The Fisher Exact Test was performed using the fisher_exact function from the scipy Python library^6^. The False Discovery Rate (Benjamini and Hochberg, 1995) was controlled per species and time-window at a *p*-value of < 0.05. This comparative analysis was performed to identify ortholog groups containing re-scheduled gene expression in CAM species in comparison with C_3_ species, in which the *S. lycopersicum* and *A. thaliana* gene were enriched in the same time-window whereas the *K. fedtschenkoi* gene was enriched in the opposite time-window (i.e., dawn vs. dusk).

## Results

### Identification of Stomata-Related Genes

**FIGURE 2:**
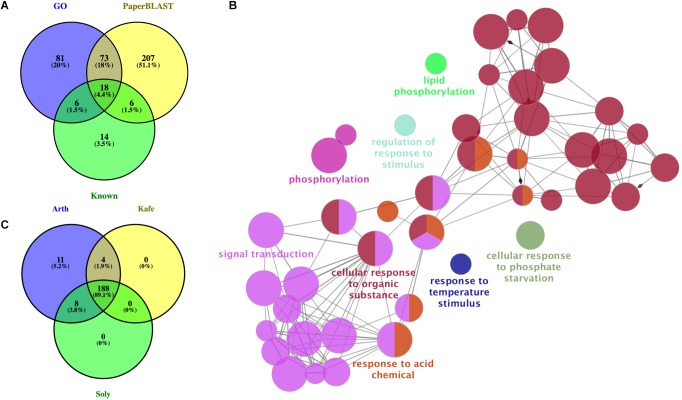
Assessment and identification of new stomata-related genes. **(A)** Overlap of *Arabidopsis thaliana* genes with annotations related to stomata, known as key stomatal genes, and identified in PaperBLAST database as stomata-related. GO: genes annotated as stomata-related from Gene Ontology analysis. Known: genes known as stomata-related from the literature. PaperBLAST: stomata-related genes identified by using PaperBLAST search. **(B)** Gene ontology enrichment of the 207 new *A. thaliana* stomatal genes. **(C)** Overlap of ortholog groups between new stomata-related genes in *A. thaliana* (Arth), *Solanum lycopersicum* (Soly), and *Kalanchoë fedtschenkoi* (Kafe).

Our PaperBLAST analysis of the protein sequences in 13 plants species identified an average of 5,196 proteins per species that could be involved in stomata-related processes, accounting for 16.4% of the respective plant genome on average (Supplementary Table S3). A total of 321 proteins in the PaperBLAST database had data containing the keywords “stomata” and “guard cell” (Table 1). Most of these proteins (304) were *A. thaliana* proteins, with the second most (9) belonging to *S. lycopersicum*. Only *A. thaliana* and *S. lycopersicum* had proteins that matched to all 321 proteins in the PaperBLAST database (Supplementary Table S3). Each protein in PaperBLAST is linked to a publication(s) that contains information on the respective protein. 272 publications were cataloged containing at least one of the 321 proteins (Supplementary Table S3). Since a majority of the 321 proteins were *A. thaliana* proteins and that *A. thaliana* is a well-annotated organism, annotations of the 304 *A. thaliana* proteins were examined. Using the current annotation of *A. thaliana* proteins, 178 proteins were annotated with stomata- or guard cell-related terms. An additional 20 genes known to play key roles in stomatal movement and development were added as well (Ohashi-Ito and Bergmann, 2006; MacAllister et al., 2007; Pillitteri et al., 2007; Kanaoka et al., 2008; Kollist et al., 2014), totaling 198 stomata-related proteins with functional annotation either GO or are known as key stomatal-related genes (Supplementary Table S4). Among the 304 *A. thaliana* stomata-related proteins in the PaperBLAST database, only 97 proteins were annotated as stomata-related in the GO database or described as key stomatal-related genes, indicating that there are 207 stomata-related genes missed by GO and hidden in the scientific literature (Supplementary Table S5 and Figure 2A). These 207 genes will be referred to as underexplored stomata-related genes/proteins from here on. The biological processes overrepresented in these 207 underexplored *A. thaliana* stomata-related proteins include signal transduction, phosphorylation-related processes, and several response processes (Figure 2B).

**Table 1:** Plants species with stomata-related proteins in PaperBLAST database.

Species	Total count of hit genes	Unique count of hit genes
*Arabidopsis thaliana*	226698	304
*Solanum lycopersicum*	6531	9
*Glycine max*	3548	1
*Oryza sativa* Japonica Group	3283	3
*Vitis vinifera*	775	2
*Zea mays*	263	2
Total		321


PaperBLAST uses an *E*-value threshold of 1e-3 to infer homology between two proteins, and generally, this *E*-value cutoff can be used reliably for this purpose (Pearson, 2013). However, to produce a more confident set of stomata-related genes for each species, the ortholog groups (OGs) of the 321 proteins linked to stomata-related processes in PaperBLAST were extracted. In the construction of these OGs, Yang et al. (2017) used a more stringent *E*-value threshold of 1e-5 to infer homology and included proteins from 26 plant species. A total of 257 OGs were extracted for the 321 proteins linked to stomata-related processes in PaperBLAST. Individually, the number of proteins from each species that shared the same OG as the PaperBLAST protein ranged from just over 500 proteins in *A. trichopoda* to over 1,200 proteins in both *A. thaliana* and *Musa acuminata* (Supplementary Table S6). Further categorization of these OGs on whether the respective species’ proteins were orthologous to an *A. thaliana* protein in any of the three stomata gene categories (i.e., annotated as stomata- /guard cell-related, known key stomatal genes, or underexplored stomata-related genes) is shown in Supplementary Table S7.

### Rescheduled Diel Expression of Stomatal Genes in *K. fedtschenkoi*

It is hypothesized that the rewiring of diel gene expression pattern played a role in the evolution of CAM plants from C_3_ plants (Yang et al., 2017). To determine if any of the *K. fedtschenkoi* genes orthologous to an *A. thaliana* gene in any of the three stomata gene categories could play a role in the inversion of day/night stomatal movement pattern, their diel expression profiles were compared to their ortholog’s expression profiles in *S. lycopersicum* and *A. thaliana*. Specifically, the gene expression profiles of 188 OGs, which were selected as they contained stomata-related genes identified from the previous section in *K. fedtschenkoi, S. lycopersicum*, and *A. thaliana* (Figure 2C).

Two criteria were used to identify stomata-related genes that display rescheduled (dawn vs. dusk) gene expression in the CAM photosynthesis species (*K. fedtschenkoi*) in comparison with the two C_3_ photosynthesis species (*S. lycopersicum* and *A. thaliana*): (1) The *K. fedtschenkoi* gene must be significantly enriched in either dawn or dusk, whereas the *S. lycopersicum* and *A. thaliana* orthologs must be significantly enriched in the opposite time-window (i.e., dawn vs. dusk), and (2) the *K. fedtschenkoi* gene must have a spearman rank correlation coefficient < -0.6 with both the *S. lycopersicum* and *A. thaliana* orthologs, whereas the *S. lycopersicum* and *A. thaliana* orthologs must have a Spearman rank correlation coefficient > 0.6 among themselves. Based on these two criteria, 16 OGs were identified to contain stomata-related genes showing rescheduled (dawn vs. dusk) gene expression in the CAM photosynthesis species in comparison with the two C_3_ photosynthesis species (Table 2 and Supplementary Figure S2). Among these 16 stomata-related OGs, only two OGs contained *A. thaliana* genes annotated as stomata-related genes: CIPK23 (AT1G30270) and RCAR3 (AT5G53160) (Table 2). The remaining 14 OGs contained *A. thaliana* genes that are not annotated or known as key stomata-related genes but have been reported to be involved in a stomata-related process in the literature, or in other words, underexplored stomata-related genes.

**Table 2:** Orthologous genes in *Arabidopsis thaliana, Solanum lycopersicum*, and *Kalanchoë fedtschenkoi* that displayed differential enrichment between dusk and dawn in gene expression.

*A. thaliana* gene name	Description	*A. thaliana* locus	*S. lycopersicum* locus	*K. fedtschenkoi* locus
ACA.l	Autoinhibited Ca2+/ATPase II	AT1G13210	Solyc01g011100	Kaladp0043s0103
AT1G17500	ATPase E1-E2 type family protein/haloacid dehalogenase-like hydrolase family protein	AT1G17500	Solyc01g096930	Kaladp0050s0103
AT1G26130	ATPase E1-E2 type family protein/haloacid dehalogenase-like hydrolase family protein	AT1G26130		
AT1G72700	ATPase E1-E2 type family protein/haloacid dehalogenase-like hydrolase family protein	AT1G72700		
PDR4	Pleiotropic drug resistance 4	AT2G26910	Solyc05g053570	Kaladp0058s0071
PDR6	Pleiotropic drug resistance 6	AT2G36380	Solyc05g055330	Kaladp0068s0280
			Solyc06g065670	Kaladp0322s0001
KCS2	3-ketoacyl-CoA synthase 2	AT1G04220	Solyc05g013220	Kaladp0020s0110
KCS8	3-ketoacyl-CoA synthase 8	AT2G15090	Solyc12g006820	Kaladp0029s0057
				Kaladp0050s0301
				Kaladp0062s0076
				Kaladp0095s0482
AT1G72180	Leucine-rich receptor-like protein kinase family protein	AT1G72180	Solyc02g091860	Kaladp0062s0167
AT5G25930	Kinase family with leucine-rich repeat domain-containing protein	AT5G25930	Solyc09g064520	Kaladp0090s0003
			Solyc12g098100	
RK1	Receptor kinase 1	AT1G65790	Solyc04g077370	Kaladp0095s0362
			Solyc04g077390	Kaladp0266s0009
CIPK23	CBL-interacting protein kinase 23	AT1G30270	Solyc01g008850	Kaladp0053s0051
CIPK3	CBL-interacting protein kinase 3	AT2G26980	Solyc12g009570	
HXK2	Hexokinase 2	AT2G19860	Solyc03g121070	Kaladp0037s0285
				Kaladp0064s0085
CPK4	Calcium-dependent protein kinase 4	AT4G09570	Solyc10g081740	Kaladp0092s0084
			Solyc11g065660	
NAK	Protein kinase superfamily protein	AT5G02290	Solyc05g053930	Kaladp0058s0603
			Solyc06g005500	
PMR6	Pectin lyase-like superfamily protein	AT3G54920	Solyc03g111690	Kaladp0024s0371
			Solyc05g014000	
RCAR1	Regulatory component of ABA receptor 1	AT1G01360	Solyc08g082180	Kaladp0042s0353
RCAR3	Regulatory components of ABA receptor 3	AT5G53160		
CNGC5	Cyclic nucleotide gated channel 5	AT5G57940	Solyc03g114110	Kaladp0008s0414
CLC-B	Chloride channel B	AT3G27170	Solyc02g094060	Kaladp0011s0070
ATML1	Homeobox-leucine zipper family protein / lipid-binding START domain-containing protein	AT4G21750	Solyc10g005330	Kaladp0093s0030
NAP5	Non-intrinsic ABC protein 5	AT1G71330	Solyc12g044820	Kaladp0040s0675
CAT2	Catalase 2	AT4G35090	Solyc02g082760	Kaladp0001s0016


### Evolutionary Dynamics of Stomata-Related Genes

The three plant species studied here have undergone multiple rounds of whole-genome duplication (Yang et al., 2017), which can cause multiple copies (i.e., paralogs) of one gene. At large, gene duplications have contributed to the evolution of novel functions (e.g., adaptation to stress) via the subsequent dynamic events that can occur after gene duplication, such as subfunctionalization and neofunctionalization (Yang et al., 2006; Panchy et al., 2016). To better understand the evolutionary dynamics that supported CAM arising from C_3_, the phylogenetic relationships within the 16 OGs were examined. Using *A. trichopoda*, which is a basal angiosperm plant species (Albert et al., 2013), as an outgroup, phylogenetic trees were constructed for each OG.

The OG containing the *A. thaliana* RCAR3 gene also included two *A. thaliana* paralogs identified as RCAR1 (AT1G01360) and RCAR2 (AT4G01026). Only two genes from *K. fedtschenkoi* and *S. lycopersicum* each were placed in this OG. Two subclades were identified in this OG, one containing the *A. thaliana* paralogs RCAR1 and RCAR2 and a *S. lycopersicum* gene (SL08G082180) and the other subclade containing the *A. thaliana* RCAR3 gene, a *S. lycopersicum* gene (SL03G007310), and the two *K. fedtschenkoi* genes (Kaladp0008s0082 and Kaladp0042s0353) (Supplementary Figure S3). RCAR genes are known to function as ABA receptors and mediate ABA-dependent regulation of type 2C protein phosphatases (Ma et al., 2009). In the presence of ABA, RCAR genes inhibit type 2C protein phosphatases phosphatase activities, which allows SNF1-related protein kinase 2 protein kinases to activate components involved in regulating stomatal movement (Culter et al., 2010; Raghavendra et al., 2010). The *K. fedtschenkoi* RCAR3 gene was identified as having rescheduled gene expression relative to the *A. thaliana* RCAR3 but not the *S. lycopersicum* RCAR3 (Supplementary Figure S3). Previous work examining the temporal differences in gene expression between the CAM species *Agave Americana* and *A. thaliana* found similar evidence of rescheduled gene expression between RCAR3 orthologs (Abraham et al., 2016). Determining the specific roles of each RCAR gene in ABA signaling, particularly in CAM species, is needed to better understand the differences in their gene expression between CAM and C_3_ species and the impact the differences could have on stomatal movement.

The phylogenetic tree of the OG containing the *A. thaliana* powdery mildew resistant 6 (PMR6, AT3G54920) gene displayed rich evolutionary dynamics as multiple genes from each of the three species were found within this OG (Supplementary Figure S4). The rescheduled genes identified in this study were not placed near each other in an evolutionary sense as the *S. lycopersicum* genes (SL03G111690, SL05G014000) were found several branching events away from the *A. thaliana* (AT3G54920) and *K. fedtschenkoi* (Kaladp0024s0371) genes. However, the *A. thaliana* and *K. fedtschenkoi* genes were closely related to each other, as well as to one *A. thaliana* (AT5G04310) and *K. fedtschenkoi* (Kaladp0008s0911) gene each and three *S. lycopersicum* genes (SL03G071570, SL05G055510, and SL11G008140). Kaladp0024s0371, identified as rescheduled relative to the *A. thaliana* PMR6 gene, is predominantly expressed during the day, while the *A. thaliana* PMR6 gene is predominantly expressed toward the end of night time (Supplementary Figure S4). The remaining closely related genes displayed similar expression covering approximately the same time window as the *A. thaliana* PMR6 gene. PMR6 encodes a pectate lyase-like protein and mutations in *PMR6* have shown to alter the composition of the plant cell wall via increases in pectin (Vogel, 2002). Moreover, stomata open more in *pmr6* mutants than in wild-type likely due to the increased cell wall stiffness of the guard cell (Woolfenden et al., 2017). Whether alteration of cell wall composition during the day contributed to the inversion of stomatal movement seen in CAM plants cannot be determined here. Further investigation into the temporal dynamics of cell wall composition in the guard cells of CAM plants would provide better clues to the role of PMR6 in stomatal movement, and to guard cell physiology in CAM plants as a whole.

**FIGURE 3:**
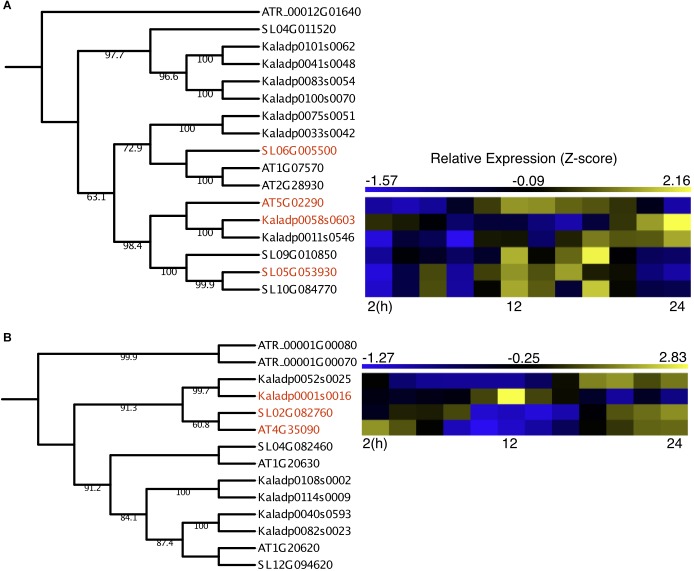
Rescheduled diel expression of stomata-related genes in a CAM photosynthesis species (*K. fedtschenkoi*) in comparison with two C_3_ photosynthesis species (*A. thaliana* and *S. lycopersicum*). **(A)** Phylogenetic tree of protein kinase genes and expression heatmaps of NAK genes in *A. thaliana, S. lycopersicum*, and *K. fedtschenkoi*. **(B)** Phylogenetic tree of catalase genes and expression heatmaps of CAT2 genes in *A. thaliana, S. lycopersicum*, and *K. fedtschenkoi*. Gene names in red text indicate genes found to be enriched in either dawn or dusk.

Two OGs containing underexplored genes had outgroup rooted trees confidently constructed, with one OG containing protein kinases and the other containing catalases. The protein kinase OG contained two *A. thaliana* genes reported to be involved in light-activation of stomatal opening, *APK1a* (AT1G07570) and *APK1b* (AT2G28930) (Elhaddad et al., 2014) and a third reported to be involved in ABA-induced osmotic stress response, *NAK* (AT5G02290) (Kodama et al., 2009). The *A. thaliana NAK* gene formed a subclade with three *S. lycopersicum* genes and two *K. fedtschenkoi* genes (Figure 3A), potentially representing duplication events after divergence from a common ancestor. The *K. fedtschenkoi NAK* gene, Kaladp0058s0603 (*NAK1*), was found to have rescheduled expression in comparison with the *A. thaliana NAK* gene, and one of the *S. lycopersicum NAK* genes (SL06G010850). The remaining *NAK* genes in *S. lycopersicum* and *K. fedtschenkoi* displayed expression covering approximately the same time window as their respective paralogs. Little information is available on the functional role of *NAK*, but Kodama et al. (2009) have reported that under drought conditions, ABA induces NAK gene expression. NAK autophosphorylates and migrates into the nucleus and phosphorylates subsequent proteins. They propose this results in modulation of nuclear function to cope with dehydration stress. *K. fedtschenkoi NAK1* peaks in the morning, while the *NAK* genes in the C_3_ plants are expressed during dusk. CAM arose as an adaptation to water-limited environments, so the rescheduled expression of an osmotic stress response gene, which is closely related to genes involved in stomatal opening, represents an interesting mode of CAM evolution.

The catalase OG contained three genes in each of the two C_3_ photosynthesis species (*A. thaliana* and *S. lycopersicum*), consistent with a previous study (Mhamdi et al., 2012). However, there were 6 *K. fedtschenkoi* genes in the catalase OG, which may result from a recent whole-genome duplication in *K. fedtschenkoi* (Yang et al., 2017). The *A. thaliana* gene in the catalase OG highlighted in Figure 3B, which contains rescheduled gene expression in the CAM species in comparison with the C_3_ species, is annotated as catalase 2 (CAT2) (Table 2). CAT2 is a part of the photorespiratory pathway and aids in the detoxification of H_2_O_2_ (Queval et al., 2007; Mhamdi et al., 2010, 2012). The CAT2 clade in the catalase OG phylogenetic tree (Figure 3B) includes all the genes listed in Table 2 for this OG but also includes another *K. fedtschenkoi* gene (Kaladp0052s0025), suggesting there are two *CAT2* genes in *K. fedtschenkoi*. The *A. thaliana* and *S. lycopersicum* genes have gene expression enriched and peaking in the morning, as well as one of the *K. fedtschenkoi CAT2* (*CAT2.1*) genes (Figure 3B). The second *K. fedtschenkoi* gene (*CAT2.2*) has gene expression enriched and peaking during dusk (Figure 3B). This presents an interesting scenario as the proteins that each catalase gene encodes for, have relatively specific roles in determining the accumulation of H_2_O_2_ produced through various metabolic pathways (Mhamdi et al., 2012).

## Discussion

Research into stomata’s role in controlling water loss and gas exchange as well as in facilitating protection against changing environmental conditions and stresses has increased recently, with the purpose of understanding the molecular mechanisms behind these features. However, the functional information generated from these studies is either missing from annotation databases or is hidden in the scientific literature (Price and Arkin, 2017), therefore, hindering the progress in functional genomics research to gain a deep and comprehensive understanding of stomata-related genes. To help alleviate this problem, this study identified genes that were neither annotated nor known as key stomata-related genes across 13 plant species, but have been reported as being involved in a stomata-related process in the literature. To demonstrate the utility of this new resource for stomata-related genes, the molecular mechanism behind the inversion of stomatal movement in CAM species, relative to C_3_ species, was investigated using the gene sets generated for one CAM species (*K. fedtschenkoi*) and two C_3_ species (*A. thaliana* and *S. lycopersicum*). Several genes were identified as candidates for further investigation into inverted stomatal movement in CAM.

A little over 300 genes were found in the literature to be involved in stomata-related processes, with a majority of them belonging to *A. thaliana*. Stomata-related genes were identified in each of the 13 plant species used in this study using PaperBLAST. The subset of genes for each species were further categorized based on sequence similarity with *A. thaliana* genes found with PaperBLAST, which were either previously annotated/known stomata-related genes, or underexplored stomata-related genes. The underexplored stomata-related genes reported in this study would serve as an excellent resource for future investigations into the molecular mechanisms behind stomata-related processes, particularly in non-Arabidopsis species. The genes identified in the 12 plant species other than *A. thaliana* could have other functional roles, even though they belong in the same ortholog group. For instance, it has been recently reported that orthologous genes involved in stomatal development and patterning in *B. distachyon* and *A. thaliana* display divergence in stomata-related function and regulation, even though they are orthologous genes (Raissig et al., 2016, 2017).

To investigate the molecular mechanism(s) that could help explain the inversion of stomatal movement seen in CAM plants, relative to C_3_ plants, the newly generated list of homologous stomata-related genes across 13 plant species was examined. CAM plants close their stomata during the day to reduce their rate of transpiration, thus enabling them to better tolerate drought-stress than C_3_ plants. ABA plays an essential role in a plant’s response to drought by facilitating stomatal closure (Vishwakarma et al., 2017), and in CAM plants, ABA concentrations have been altered to peak right before morning (Abraham et al., 2016). The *NAK* gene has been characterized to be induced by ABA in drought conditions (Kodama et al., 2009) and was identified in the list of homologous stomatal-related genes as having rescheduled gene expression between *K. fedtschenkoi* and two C_3_ species. Assuming the *NAK1* gene in *K. fedtschenkoi* has the same function as its *A. thaliana* ortholog, it can be hypothesized that the shift in its expression could likely result from or result in the change in ABA concentrations. This hypothesis can be tested using molecular genetics and functional approaches in the future (e.g., characterization of knockout *nak1* mutants in *K. fedtschenkoi*). Determining the role of *NAK1* in *K. fedtschenkoi*, and how it relates to stomatal movement, could provide new insights into the molecular mechanisms which CAM plants use to adapt to a dry environment. Moreover, identifying the regulator of *NAK1* could also help in understanding ABA signaling, and therefore stomatal closing, in CAM plants.

**FIGURE 4:**
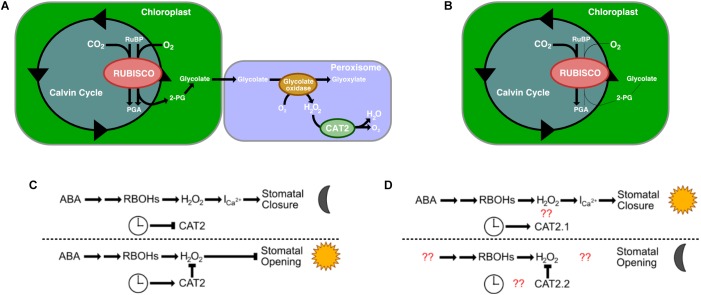
The role of Catalase 2′s (CAT2) roles in C_3_ plants and in CAM plants. **(A)** CAT2’s role in photorespiration in C_3_ plants. **(B)** Photorespiration is reduced in CAM plants. **(C)** CAT2’s role in stomatal open closing (top) and opening (bottom) in C_3_ plants. **(D)** CAT2’s predicted role in stomatal closing (top) and opening (bottom). Red question marks indict unknown interactions that warrant further investigation. RuBP, Ribulose 1,5-biphosphate; PGA, 3-phosphoglyceric acid; 2-PG, 2-phosphogylycolate; I_ca2+_, Ca^2+^ channels; H_2_O_2_, hydrogen peroxide; RBOH, respiratory burst oxidases.

An additional gene identified in the list of homologous stomatal-related genes as having undergone rescheduling of gene expression in *K. fedtschenkoi* relative to C_3_ species was a catalase gene, CAT2. There are generally three catalase genes in a plant’s genome, and each gene is considered functionally conserved between species (Mhamdi et al., 2012). Specific to the CAT2 gene, CAT2 is expressed in photosynthetic mesophyll cells and guard cells, is involved in photorespiration, and shows day-night rhythms in transcript abundance, with peaks in the morning (Zhong et al., 1994; Zhong and McClung, 1996; Queval et al., 2007; Mhamdi et al., 2010; Mhamdi et al., 2012). CAT2’s primary role is in H_2_O_2_ detoxification, specifically H_2_O_2_ produced as a result of photosynthesis (Mhamdi et al., 2010). Rubisco is a bifunctional enzyme that also catalyzes oxygenation of RuBP, which produces 2-phosphogylycolate as one of its products. This small molecule is not metabolized via the Calvin-Benson cycle but is dephosphorylated to produce glycolate, which is transported to the peroxisomes. Within the peroxisomes, glycolate is oxidized to glyoxylate using oxygen as a co-factor, which results in H_2_O_2_ being produced. This production is negatively controlled by CAT2 (Figure 4A).

CAM is a mechanism to reduce photorespiration by reducing the CO_2_:O_2_ ratio in cells (Figure 4B). In other organisms that have evolved a method to reduce glycolate production, the main fate of glycolate is excretion or oxidation to glyoxylate via a mitochondrial dehydrogenase using NAD^+^ as the final electron acceptor (Stabenau and Winkler, 2005). Whether this is the case in *K. fedtschenkoi* is yet to be determined, however, our results suggest a reduced role for CAT2.1 in *K. fedtschenkoi*. Interestingly, CAT2 seems to be duplicated in *K. fedtschenkoi*, with one copy having conserved gene expression and the other copy being expressed at dawn (Figure 3B). In a study in *A. thaliana*, CAT2 was found to be involved in ABA signaling for stomatal closure as *cat2* mutants had significantly enhanced ABA-induced stomatal closure (Jannat et al., 2011). These authors presented a model where ABA activates respiratory burst oxidases (RBOHs) in the membranes of guard cells which results in rapid production of H_2_O_2_ in the guard cells (Kwak et al., 2003). The H_2_O_2_ then activates Ca^2+^ channels causing stomatal closure during the night (Pei et al., 2000; Murata et al., 2001; Figure 4C). CAT2 plays a negative role in ABA signaling in stomatal closure by detoxifying the guard cells. This results in Ca^2+^ channels not being activated which leaves the stomata open (Figure 4C). Due to the role of CAT2 in stomatal opening, we propose that CAT2.2, expressed during dusk in *K. fedtschenkoi*, could play a role in stomatal opening by inhibiting ABA-induced stomatal closure by reducing H_2_O_2_ concentrations (Figure 4D). Several other authors have suggested that H_2_O_2_ activation of Ca^2+^ channels represents a possible convergent point for multiple stress signaling pathways (Gechev and Hille, 2005; Sewelam et al., 2014; Niu and Liao, 2016). Functional characterization of both CAT2 genes in *K. fedtschenkoi* is needed to determine any roles in stomatal opening, as well as determine if CAT2.2 is circadian regulated. Further determination of the role of H_2_O_2_ signaling in *K. fedtschenkoi* stomatal movement is needed.

In summary, this study cataloged hundreds of underexplored stomata-related genes in multiple plant species including C3, C4, and CAM photosynthesis plants. We also identified numerous underexplored stomata-related genes that displayed re-scheduled gene expression between orthologs in a CAM species and two C_3_ species, providing valuable candidates for CAM-engineering in C3 photosynthesis crops to enhance drought-resistance. Furthermore, the impact of gene duplication and diversification on CAM evolution was underlined, highlighting the evolutionary dynamics involved in CAM evolution.

## Author Contributions

RM and XY conceived the research. RM performed all data analyses and wrote the manuscript. GT and XY provided input during the study and edited the manuscript.

## Conflict of Interest Statement

The authors declare that the research was conducted in the absence of any commercial or financial relationships that could be construed as a potential conflict of interest.

## Footnotes

1 https://www.ebi.ac.uk/Tools/msa/muscle/

2 http://www.iqtree.org/

3 https://phytozome.jgi.doe.gov/pz/portal.html

4 https://www.ddbj.nig.ac.jp/index-e.html

5 https://pandas.pydata.org/

6 https://www.scipy.org/
