# Supplementary material for: Comparative Genomics Analysis Provides New Insight Into Molecular Basis of Stomatal Movement in Kalanchoë fedtschenkoi
Source: Front Plant Sci. 2019 Mar 13;10:292. doi: 10.3389/fpls.2019.00292 (PMC6425862; doi:10.3389/fpls.2019.00292)
Supplement: Supplementary file 3 [file Data_Sheet_1.docx]

Supplementary Material

Comparative Genomics Analysis Provides New Insight into Molecular Basis of Stomatal Development and Movement in *Kalanchoë fedtschenkoi*

Robert C. Moseley, Gerald A. Tuskan, and Xiaohan Yang^*^

*** Correspondence:** Dr. Xiaohan: yangx@ornl.gov

# Supplementary Figures and Tables

## Supplementary Figures

##
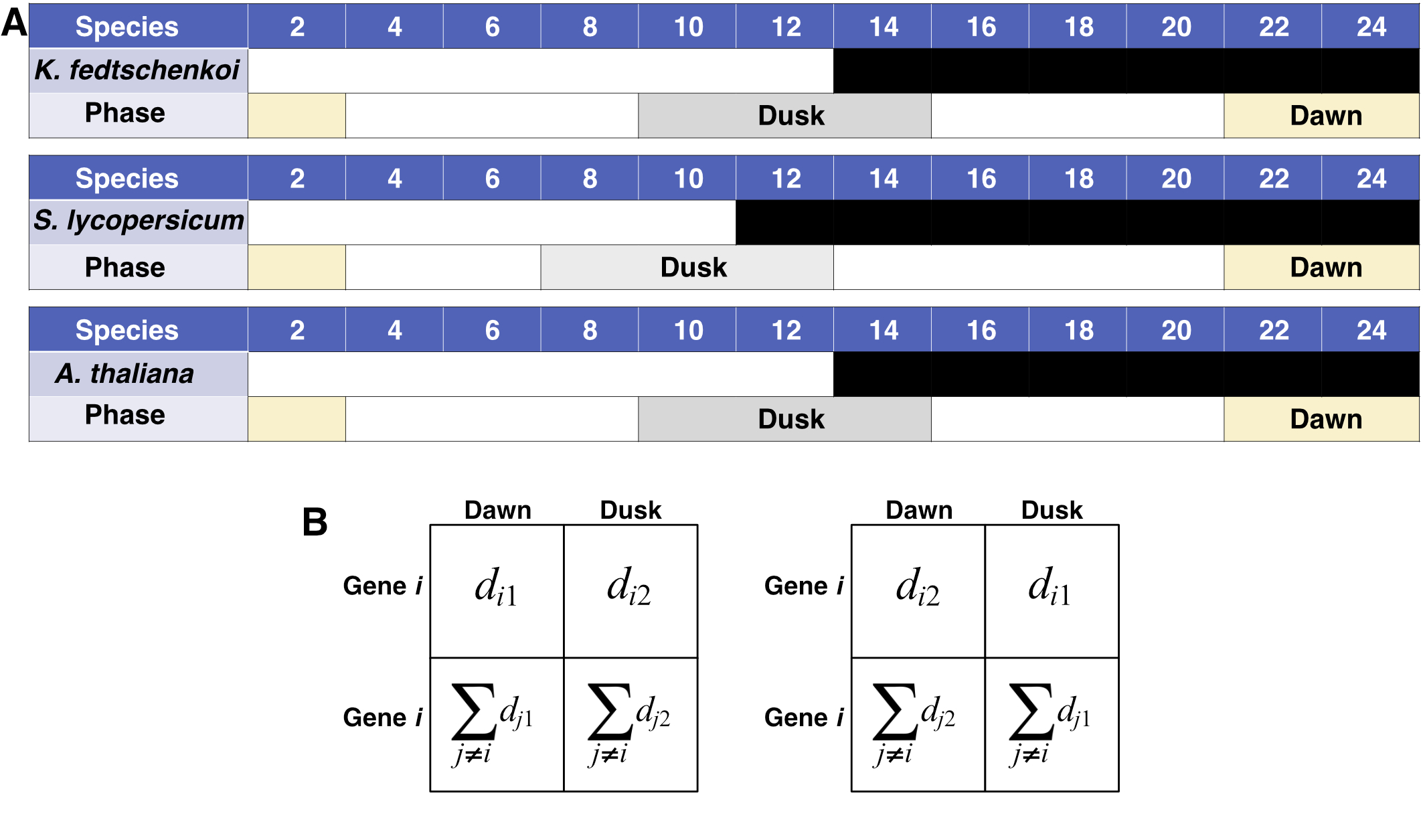


**Supplementary Figure S1. Description of dawn and dusk phases for each species and the contingency tables used to gene expression enrichment.**

**A)** The time points from each species’ time-course data that were considered as occurring during dusk and dawn. **B)** Contingency tables, adapted from Yang et al., (2017), for Fisher Exact Test to determine gene expression enrichment between dusk and dawn.


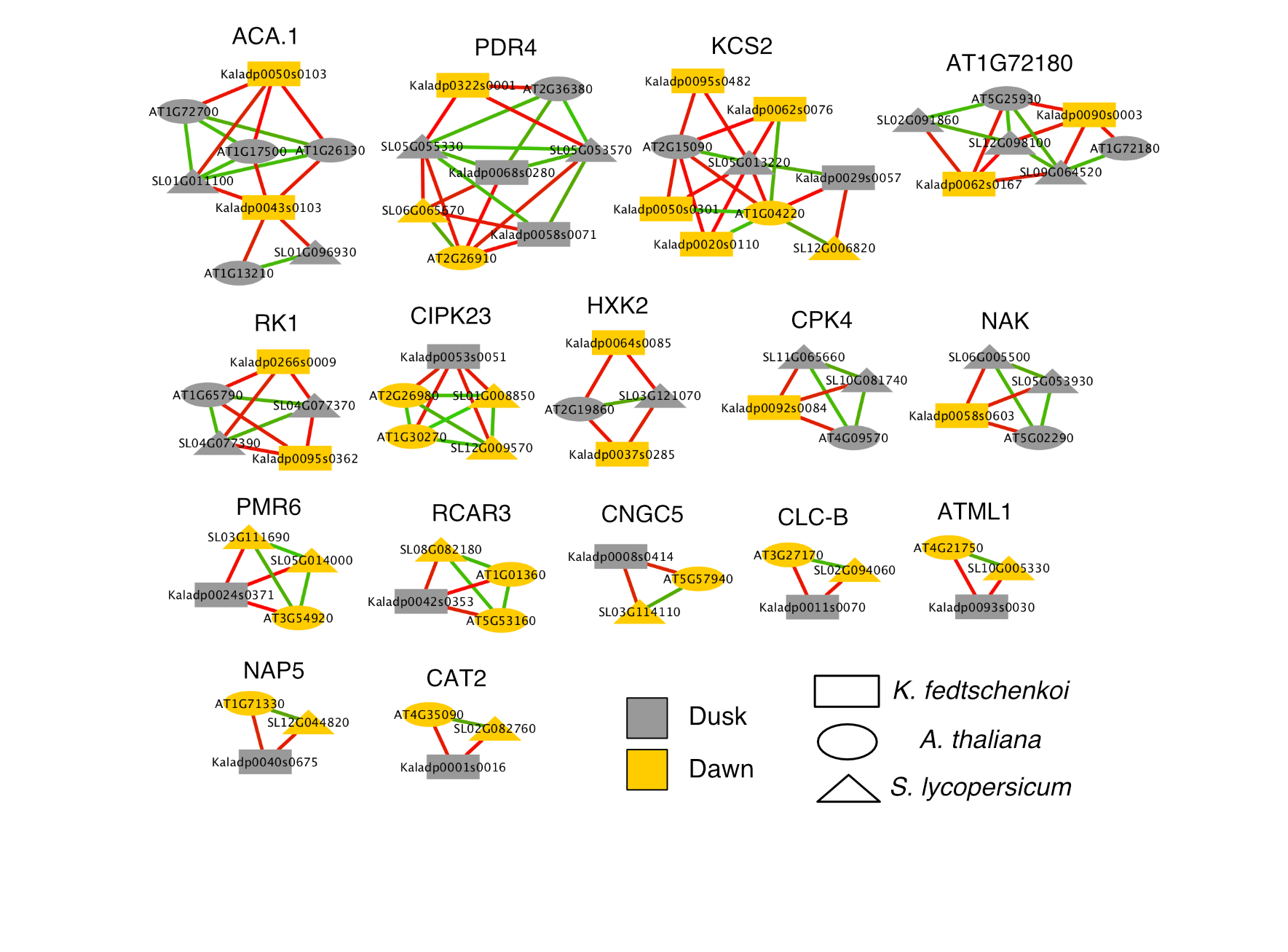


**Supplementary Figure S2. Gene clusters of genes with expression enriched in either dusk or dawn.**

Genes in ortholog groups defined as rescheduled. Green edges represent rho ≥0.6 and red edges represent rho ≤-0.6. Rectangular, oval, and triangular nodes represent genes belonging to *Kalanchoë fedtschenkoi*, *Arabidopsis thaliana*, and *Solanum lycopersicum*, respectively. Nodes colored grey or yellow are genes that are enriched in dusk or dawn, respectively.


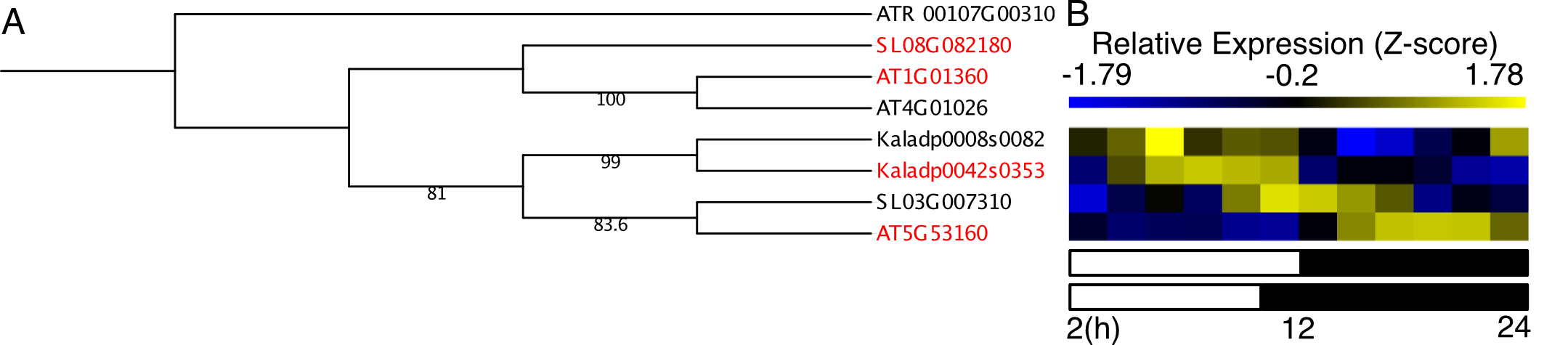


**Supplementary Figure S3. Rescheduled diel expression of RCAR genes in a CAM photosynthesis species (*Kalanchoë fedtschenkoi*) in comparison with two C_3_ photosynthesis species (*Arabidopsis thaliana*, *Solanum lycopersicum*).**

**A**) Phylogenetic tree of RCAR genes. Gene names in red font indicate genes found to be enriched in either dawn or dusk. **B**) Expression heatmaps of RCAR3 genes in *A. thaliana*, *S. lycopersicum*, and *K. fedtschenkoi*. White and black bars indicate daytime (12-hour for *A. thaliana* and *K. fedtschenkoi*; 10-hour for *S. lycopersicum*) and nighttime (12-hour for *A. thaliana* and *K. fedtschenkoi*; 14-hour for *S. lycopersicum*), respectively.


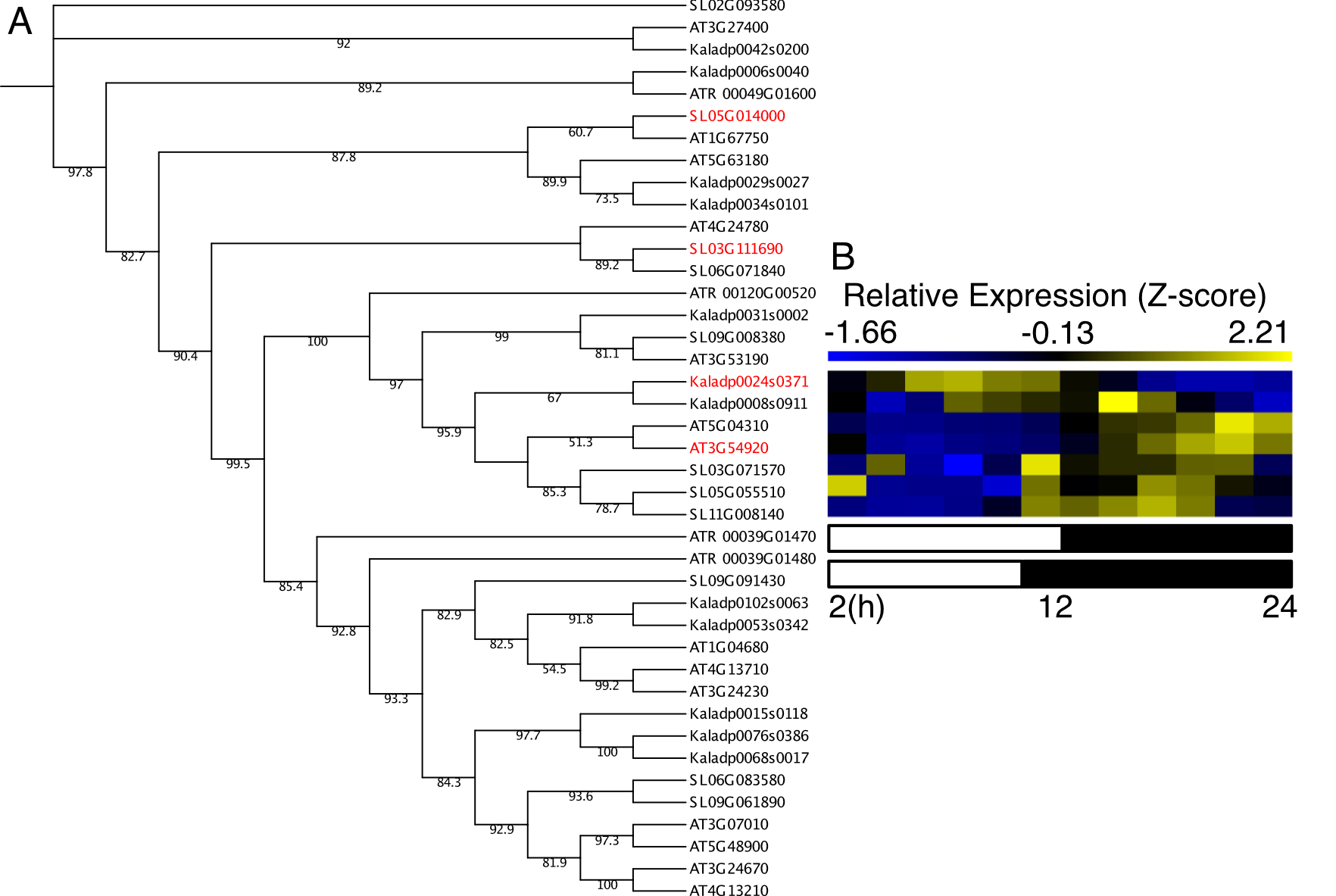


**Supplementary Figure S4. Rescheduled diel expression of PMR genes in a CAM photosynthesis species (*Kalanchoë fedtschenkoi*) in comparison with two C_3_ photosynthesis species (*Arabidopsis thaliana*, *Solanum lycopersicum*).**

**A**) Phylogenetic tree of PMR genes. Gene names in red font indicate genes found to be enriched in either dawn or dusk. **B**) Expression heatmaps of PMR6 genes in *A. thaliana*, *S. lycopersicum*, and *K. fedtschenkoi*. White and black bars indicate daytime (12-hour for *A. thaliana* and *K. fedtschenkoi*; 10-hour for *S. lycopersicum*) and nighttime (12-hour for *A. thaliana* and *K. fedtschenkoi*; 14-hour for *S. lycopersicum*), respectively.

## Supplementary Tables

**Supplementary Table S1**. **Plant species used in PaperBLAST search.**

| Species Abbrev. | Species Name | Version | Data Provider | PubMed ID |
| --- | --- | --- | --- | --- |
| arth | *Arabidopsis thaliana* | Araport11 | Plaza 4.0 | 27862469 |
| vivi | *Vitis vinifera* | 12xMarch2010 | Plaza 4.0 | 17721507 |
| soly | *Solanum lycopersicum* | itag2.4 | Plaza 4.0 | 22660326 |
| migu | *Mimulus guttatus* | v2.0 | Phytozome v12 | 17460045 |
| kafe | *Kalanchoë fedtschenkoi* | v1.1 | Phytozome v12 | 29196618 |
| sobi | *Sorghum bicolor* | v3.1 | Plaza 4.0 | 19189423 |
| seit | *Setaria italica* | v2.2 | Plaza 4.0 | 22580950 |
| brdi | *Brachypodium distachyon* | v3.1 | Plaza 4.0 | 20148030 |
| orsaja | *Oryza sativa ssp. japonica* | v7.0 | Plaza 4.0 | 16100779 |
| anco | *Ananas comosus* | v3.0 | Plaza 4.0 | 26523774 |
| muac | *Musa acuminata* | v1.0 | Plaza 4.0 | 22801500 |
| pheq | *Phalaenopsis equestris* | v1.0 | Plaza 4.0 | 25420146 |
| amtr | *Amborella trichopoda* | JGI v1.0 | Plaza 4.0 | 24357323 |

**Supplementary Table S2. PaperBLAST results filtered on keywords ‘stomata’ and ‘guard cell’.** (*Separate file*)

**Supplementary Table S3**. **Number of** **stomata-related proteins identified in 13 plant species using PaperBLAST.**

The full names of the plant species are listed in Supplementary Table S2.

| Item | Plant Species | | | | | | | | | | | | | Average |
| --- | --- | --- | --- | --- | --- | --- | --- | --- | --- | --- | --- | --- | --- | --- |
|  | Amtr | Anco | Arth | Brdi | Kafe | Migu | Muac | Orsa | Pheq | Seit | Sobi | Soly | Vivi |  |
| # of Proteins^a^ | 26846 | 27024 | 27615 | 34310 | 30964 | 28140 | 36528 | 42189 | 29415 | 34584 | 34211 | 34725 | 26346 | 31761 |
| Total Filtered Proteins^b^ | 12205 | 14724 | 17537 | 17464 | 19563 | 18914 | 27326 | 22094 | 13800 | 20780 | 19081 | 19798 | 17812 | 18546 |
| Total Unique Proteins^c^ | 3647 | 4146 | 5197 | 4953 | 5595 | 5649 | 6956 | 5936 | 3998 | 5718 | 5371 | 5572 | 4813 | 5196 |
| % of Genome^d^ | 0.136 | 0.153 | 0.188 | 0.144 | 0.181 | 0.201 | 0.19 | 0.141 | 0.136 | 0.165 | 0.157 | 0.16 | 0.183 | 0.164 |
| Total Unique Hit Proteins^e^ | 318 | 330 | 318 | 319 | 320 | 319 | 317 | 319 | 318 | 318 | 318 | 321 | 319 | 320 |
| Total Unique Publications^f^ | 270 | 268 | 271 | 270 | 271 | 271 | 269 | 271 | 270 | 270 | 270 | 271 | 271 | 270 |

^a^Total number of proteins in a plant species.

^b^Total number of times a protein in a plant species matched on sequence to a protein in the PaperBLAST database that was associated with stomata or guard cell.

^b^Total number of proteins in a plant species after filtering out multiple matches to a protein in the PaperBLAST.

^d^Percent of genome that the proteins in “Total Unique Proteins” represent.

^e^Total number of proteins in PaperBLAST that matched to a protein in a plant species.

^f^Total number of publications associated with a PaperBLAST protein that matched to a protein in a plant species.

**Supplementary Table S4. *Arabidopsis thaliana* genes with stomata-related annotations or known as key stomatal genes.** (*Separate file*)

**Supplementary Table S5**. ***Arabidopsis thaliana* genes identified as underexplored stomata-related genes.** (*Separate file*)

**Supplementary Table S6. Categorization of whether genes in each species were placed in the same ortholog group (OG) as the gene they matched to in the PaperBLAST database.**

The full names of the plant species are listed in Supplementary Table 2.

| Species | Amtr | Arth | Brdi | Migu | Muac | Orsa | Pheq | Seit | Sobi | Soly | Vivi | Anco | Kafe |
| --- | --- | --- | --- | --- | --- | --- | --- | --- | --- | --- | --- | --- | --- |
| Same OG | 541 | 1253 | 908 | 989 | 1271 | 993 | 668 | 1020 | 895 | 986 | 821 | 716 | 1045 |
| Different OG | 6256 | 9341 | 9965 | 10609 | 13496 | 12596 | 6531 | 12177 | 10977 | 10566 | 9277 | 7732 | 10714 |
| No OG (query only) | 1415 | 749 | 878 | 1009 | 2184 | 1510 | 1607 | 922 | 1289 | 1385 | 1881 | 1062 | 777 |
| No OG (hit only) | 85 | 145 | 118 | 115 | 183 | 135 | 98 | 131 | 131 | 127 | 104 | 92 | 158 |
| No OG (both) | 13 | 21 | 6 | 17 | 42 | 16 | 26 | 8 | 12 | 15 | 26 | 22 | 18 |

**Supplementary Table S7. Categorization of genes placed in the same ortholog group as the gene they matched to in the PaperBLAST database based on whether the matched gene was annotated as stomata-related or known as a key stomatal gene.**

The full names of the plant species are listed in Supplementary Table 2.

| Category | Plant Species | | | | | | | | | | | | |
| --- | --- | --- | --- | --- | --- | --- | --- | --- | --- | --- | --- | --- | --- |
|  | Amtr | Arth | Brdi | Migu | Muac | Orsa | Pheq | Seit | Sobi | Soly | Vivi | Anco | Kafe |
| Annotated or Key orthologs | 125 | 157 | 286 | 191 | 181 | 282 | 193 | 149 | 205 | 190 | 180 | 173 | 203 |
| New Orthologs | 395 | 530 | 914 | 687 | 768 | 945 | 768 | 493 | 784 | 674 | 755 | 616 | 792 |
